# Acute Radiation Syndrome and the Microbiome: Impact and Review

**DOI:** 10.3389/fphar.2021.643283

**Published:** 2021-05-18

**Authors:** Brynn A. Hollingsworth, David R. Cassatt, Andrea L. DiCarlo, Carmen I. Rios, Merriline M. Satyamitra, Thomas A. Winters, Lanyn P. Taliaferro

**Affiliations:** Radiation and Nuclear Countermeasures Program (RNCP), Division of Allergy, Immunology and Transplantation (DAIT), National Institute of Allergy and Infectious Diseases (NIAID), National Institutes of Health (NIH), Rockville, MD, United States

**Keywords:** radiation, microbiome, radiation medical countermeasure, radiation biodosimetry, acute radiation syndrome

## Abstract

Study of the human microbiota has been a centuries-long endeavor, but since the inception of the National Institutes of Health (NIH) Human Microbiome Project in 2007, research has greatly expanded, including the space involving radiation injury. As acute radiation syndrome (ARS) is multisystemic, the microbiome niches across all areas of the body may be affected. This review highlights advances in radiation research examining the effect of irradiation on the microbiome and its potential use as a target for medical countermeasures or biodosimetry approaches, or as a medical countermeasure itself. The authors also address animal model considerations for designing studies, and the potential to use the microbiome as a biomarker to assess radiation exposure and predict outcome. Recent research has shown that the microbiome holds enormous potential for mitigation of radiation injury, in the context of both radiotherapy and radiological/nuclear public health emergencies. Gaps still exist, but the field is moving forward with much promise.

## Introduction

Understanding the role of the microbiome in radiation pathogenesis, assessment of exposure, protection, and mitigation of injury following acute radiation exposure is of great interest. Such studies may help reveal new mechanisms of action, medical countermeasures (MCMs), and biomarkers for biodosimetry to be used in the event of a radiation public health emergency. Radiation exposures resulting from environmental, accidental, medical, or terrorist radiation/nuclear incidents (e.g., improvised nuclear device or radiological dispersal device) have the potential to affect the health and function of many biological systems. The possible dose ranges and radiation sources (e.g., gamma, neutron, X-ray, and mixed-field) involved in these exposures could span nearly all conceivable scenarios, from internalized radionuclides to photons and/or particulate radiation exposure, with doses from near background to high-lethal exposures (Glasstone et al., 1977; Newbold et al., 2019). The Radiation and Nuclear Countermeasures Program (RNCP) within the National Institute of Allergy and Infectious Diseases (NIAID) of the National Institutes of Health (NIH), was initiated in 2004 following a congressional mandate to fund research to develop medical-based approaches for use after a radiological or nuclear public health incident (Hafer et al., 2010; Rios et al., 2014). As of early 2021, four products have been approved by the U.S. Food and Drug Administration (FDA) to treat hematopoietic complications following acute radiation exposure—filgrastim (Neupogen®, Amgen, FDA approved March 2015) (Food and Drug Administration, 2015a), pegfilgrastim (Neulasta®, Amgen, FDA approved November 2015) (Food and Drug Administration, 2015b), sargramostim (Leukine®, Partner Therapeutics, FDA approved March 2018) (Food and Drug Administration, 2018), and romiplostim (Nplate®, Amgen, FDA approved January 2021). However, products are yet to be approved to treat other acute or delayed subsyndromes, such as gastrointestinal (GI) or lung, nor have any radiation biodosimetry tests been cleared for triage or dose assessment. It is possible that some of these gaps could be filled as researchers dig deeper into the complexities of the human microbiome and its involvement in radiation injury. This recently renewed area of research, with a focus on the acute radiation exposure setting, could lead to exciting new drug targets, MCMs, and biomarkers of radiation injury.

## History of Microbiome Research

**FIGURE 1:**
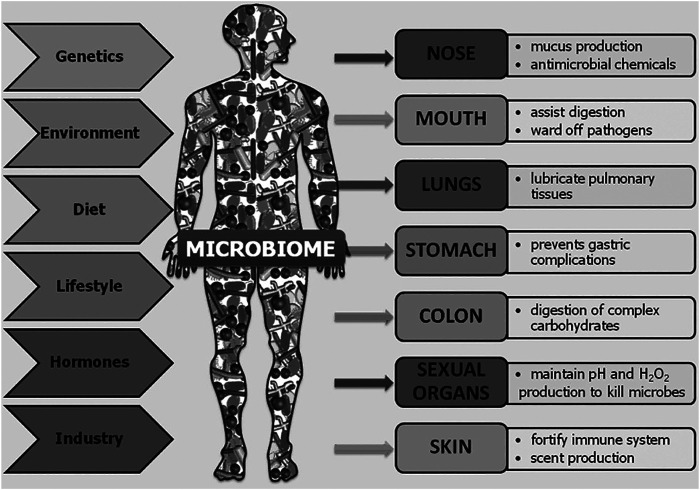
Overview of the body areas inhabited by microbiota, their roles in those organs, and the factors contributing to their diversity among individuals and across time. Reprinted from *Human Microbes—The Power Within*, by V.D. Appanna, 2018. Springer Singapore (Appanna, 2018).

It has long been known that microbes inhabit the human body alongside human cells in a symbiotic relationship. In 1886, Escherich published that *Escherichia coli* bacteria lived not only in the intestines of children with diarrheal disease but also in those of healthy children (Hayes and Sahu, 2020). Over the years, it has been determined that the human body is host to between 75 and 200 trillion microbes, similar to the total number of human cells in the body (Ursell et al., 2012; Sender et al., 2016; Sender et al., 2016; Hayes and Sahu, 2020). In 2001, Lederberg, a Nobel Prize recipient for work on microbial genetics, defined “microbiome” as “the collective genomes of all the microorganisms inhabiting a specific environment, especially that of the body” (Lederberg and McCray, 2001). Microbiota not only refers to bacteria, but encompasses all the microorganisms of the body, including archaea, fungi, protozoans, bacteria, and viruses (Lederberg and McCray, 2001; Zhu et al., 2010; Jandhyala et al., 2015). The human microbiome is incredibly diverse with an individual’s microbiome so distinct that it has been proposed to be used as a differentiating biomarker in forensics (D’Angiolella et al., 2020; García et al., 2020). Not only is the microbiome diverse among individuals but also across the body and even within body areas (Roth and James, 1988; Hakansson and Molin, 2011; Seidel et al., 2020). This diverse microbiota plays a critical role in the biological function of the gut, skin, lungs, oral cavity, urogenital system, and more (Figure 1). The microbiota occupying the organs comprises differing types and abundance of microbial species (Table 1). Microbial diversity, or lack thereof, depending on the body system examined, is also an important indicator of health (Muhleisen and Herbst-Kralovetz, 2016; Buchta, 2018; Ferreira et al., 2019; Araghi, 2020).

**TABLE 1:** Dominant bacteria in microbial communities across the human body.

Body area	Bacteria	Characterization
GI (Arumugam et al., 2011; Hakansson and Molin, 2011; King et al., 2019)	Firmicutes phylum	Together with bacteroidetes makes up 80% of the gut flora
	Bacteroidetes phylum	Together with Firmicutes makes up 80% of the gut flora
	Actinobacteria phylum	Makes up ∼3% of the gut flora
	Proteobacteria phylum	Makes up ∼1% of the gut flora
Oral Cavity (Aas et al., 2005; Bik et al., 2010)	*Veillonella*	Predominant genus across the oral cavity, in the phylum Firmicutes
	*Actinomyces*	Predominant genus on the tongue and teeth, in the phylum Actinobacteria
	*Neisseria*	Predominant genus on the lips, palate, and cheek, in the phylum Proteobacteria
	*Simonsiella*	Predominant genus on the tongue, in the phylum Proteobacteria
	*Eubacterium*	Predominant genus on the teeth, in the phylum Firmicutes
Skin (Davis, 1996)	*Staphylococcus epidermidis*	Most abundant skin inhabitant making up 90% of the resident aerobic flora
	*Micrococcus luteus*	Accounts for 20–80% of the micrococci isolated from the throughout the normal skin
	*Staphylococcus aureus*	Common location: nose, perineum, and vulvar skin. Presence varies with age. More abundant with dermatologic disease
Lung (Charlson et al., 2011; Dickson et al., 2013; Liu et al., 2020)	*Prevotella*	Makes up 7–23% of microbes from healthy subjects’ bronchoalveolar lavage, genus in the Bacteroidetes phylum
	*Veillonella*	Makes up 6–15% of microbes from healthy subjects’ bronchoalveolar lavage, genus in the phylum Firmicutes
Naso-pharyngeal (Frank et al., 2010)	*Propionibacterium acnes*	Makes up ∼42% of microbes from healthy subject nasal swabs, member of Actinobacteria phylum
	*Staphylococcus epidermidis*	Makes up ∼10% of microbes from healthy subject nasal swabs, member of Firmicutes phylum
	*Corynebacterium tuberculostearicum*	Makes up ∼8% of microbes from healthy subject nasal swabs, member of Actinobacteria phylum
Vaginal (Ravel et al., 2011; Muhleisen and Herbst-Kralovetz, 2016; Buchta, 2018)	*Lactobacillus iners*	Makes up 1–88% of healthy vaginal microbiota, with 34% of healthy females’ vaginal microbiota dominated by this species
	*Lactobacillus crispatus*	Makes up 0–83% of healthy vaginal microbiota, with 27% of healthy females’ vaginal microbiota dominated by this species
	*Lactobacillus gasseri*	Makes up 0.4–86% of healthy vaginal microbiota, with 6% of healthy females’ vaginal microbiota dominated by this species
	*Lactobacillus jensenii*	Makes up 0.5–80% of healthy vaginal microbiota, with 5% of healthy females’ vaginal microbiota dominated by this species

While these microbes take their nutrients from the human body, they contribute to the health of the human host as well. Roles include outcompeting pathogenic microbes, assisting in nutrient breakdown and metabolism, and involvement in complex interactions with the immune system (Appanna, 2018). The presence of the microbiota stimulates expression of pattern recognition receptors (Brandl et al., 2007; Gallo and Nizet, 2008; Vaishnava et al., 2008; Vaishnava et al., 2011), secretion of protective proteins like mucins (Sanford and Gallo, 2013; Pickard et al., 2017; Meisel et al., 2018), as well as immune cell production, maturation, and recruitment, particularly of regulatory T-cells (Hamada et al., 2002; Kupper and Fuhlbrigge, 2004; Paulos et al., 2007; Bouskra et al., 2008). Interestingly, some immune cells are able to discriminate between pathogenic and commensal bacteria (Franchi et al., 2012; Seneschal et al., 2012; Guo et al., 2020). In addition, there are extensive and complex interactions across the distinct microbial communities spanning the body including the so-called gut–lung axis, microbiota–gut–liver axis, and the microbiota–gut–brain axis (Keely et al., 2012; Dumas et al., 2018; Bajaj et al., 2019; Nie et al., 2020; Stavropoulou and Bezirtzoglou, 2020). Overall, the microbiome plays a vital role in human health and, in some ways, each distinct microbiota axis represents a system unto itself.

Since the initial research and visualization of cells via microscopy in the 1660s by Hooke and van Leeuwenhoek, humans have investigated microscopic organisms around and in us; and with the inception of the NIH Human Microbiome Project in 2007, research into the microbiome has exploded (Proctor et al., 2019). For most of the history of microbiome research, identification was limited to only a few hundred species that could be cultured (Lee et al., 1968; Moore and Holdeman, 1974), but with advances in whole genome sequencing, Relman and others encouraged researchers to utilize these new technologies to identify previously unrecognized, unculturable microbes that inhabit the human body (Relman, 1999; Relman, 2002). Since that time, it has been observed that 60–80% of human-colonizing bacterial species cannot be cultured with standard medical microbiology media (Suau et al., 1999). Recently, the microbial 16S ribosomal RNA (16S rRNA) gene sequencing method has been employed to conduct culture-independent investigations of microbiota composition across the body in numerous mammalian species, including humans (Muegge et al., 2011). The discovery of the 1.5-Kbp 16S rRNA gene, containing highly conserved ubiquitous sequences and regions that vary with greater or lesser frequency over evolutionary time, revolutionized culture-independent microbial determination (Lane et al., 1985; Böttger, 1989). Through this research, genus- and species-level identification and abundance across individuals and across their body regions (Table 1) have uncovered high inter-individual and intra-individual microbiota diversity that is impacted by co-evolutionary selection, age, diet, and geographic region (Mackie et al., 1999; Spor et al., 2011; Lozupone et al., 2012; Morgan and Huttenhower, 2012; Yatsunenko et al., 2012). While there is no core microbiome at the species level, at the phylum level, there is commonality and a broad consensus for similarities in functional gene profiles (Sekelja et al., 2011; Morgan et al., 2013; Sharpton, 2018).

Although the discovery and use of the 16S rRNA gene have greatly expanded microbiome research, it is still only bacterially selective, limiting this sequencing technique to evaluation of bacterial composition and responses to environmental changes or challenges (Bäckhed et al., 2005). Investigation of the virome, mycobiome, and archaea components of the microbiota broadly and particularly in response to radiation has been lacking (Rosenberg and Zilber-Rosenberg, 2013; Roy and Trinchieri, 2017; Liu et al., 2021). It is possible that broader insights into the impact of nonbacterial components of the GI microbiota might be obtained through non-targeted shotgun metagenomic sequencing techniques that would be capable of assessing radiation responses in the nonbacterial compartments of the GI microbiota (Campo et al., 2020; Kaźmierczak-Siedlecka et al., 2020; Turkington et al., 2021). The current lack of studies investigating GI microbiota compartments beyond the bacteriome represents a potentially important gap in our understanding of the impact of the microbiome on radiation response.

In this review, the effect that radiation has on the microbiota of various parts of the human body is summarized. Animal models of acute radiation exposure and their use for future microbiome studies are then discussed. Given the enormous therapeutic potential of the microbiome in mitigating multiple organ damage from irradiation (e.g., the GI tract, lung, and skin), consideration of these microbial populations in research and development is necessary. A discussion of treatments and other factors that have been shown to modify the microbiome, mitigating radiation damage, is presented. These approaches can preserve organ function and health, potentially allowing the microbiome to serve as a MCM and/or biomarker for radiation injury.

**FIGURE 2:**
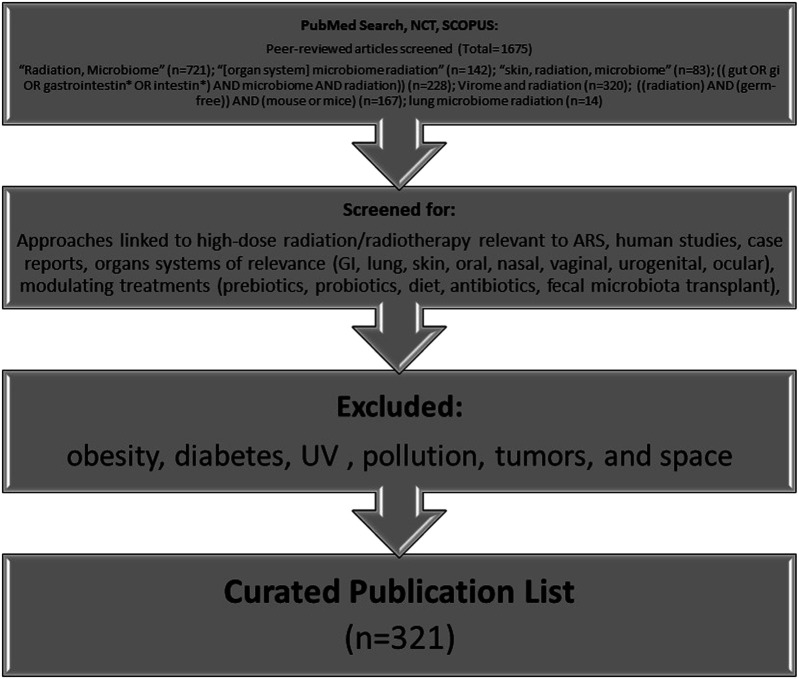
Methodology for curation of literature for this review (*NCT: clinicaltrials.gov).

To date, human microbiome studies in radiological or nuclear incidents do not exist. Thus, most radiation studies, and especially those examining the microbiome, are conducted in the context of medical treatment, primarily with respect to cancer radiotherapy. While these data are helpful for guiding future studies in the acute radiation exposure space, it is not directly comparable to an acute radiation exposure scenario. Furthermore, it is important to note that even cancer alone affects the microbiome (Nam et al., 2013), and this must be taken into consideration when extrapolating data from these studies to the context of a radiological or nuclear incident. In an effort to curate currently available data relevant to ARS, a systematic search methodology was conducted and is highlighted in Figure 2. In summary, related keywords were used to search PubMed, Scopus, and clinicaltrials.gov (trials referenced using the National Clinical Trial (NCT) number), followed by screens for approaches linked to high-dose radiation or radiotherapy relevant to ARS. In particular, research articles were selected based on organ systems of interest and treatment approaches that could modulate the microbiome. Certain areas of microbiome research (e.g., obesity, diabetes, ultraviolet (UV), pollution, tumors, space, and those unrelated to biology) were excluded.

## The Effects of Radiation on the Microbiome

### Gut Microbiome

The microbiota of the human GI tract is essential for metabolic and digestive function, development, and support of the gut-associated immune system, prevention of gut colonization by pathogenic microbial species, and support of epithelial integrity to prevent barrier translocation of microbes (Bäckhed et al., 2005; Hooper and MacPherson, 2010; Stecher and Hardt, 2011). Studies suggest that the human GI tract harbors more than 800 different individual bacterial species (Turnbaugh et al., 2010) with proportional representation, genus level distribution, and viable count of colony-forming units (CFUs) varying widely from the oral cavity to the rectum (Hayashi et al., 2005; Wang et al., 2005; Bik et al., 2006; Lazarevic et al., 2009; Hakansson and Molin, 2011) and changing with age, diet, and geographical location (Biagi et al., 2010; Claesson et al., 2011). The predominant phyla in the healthy gut are Firmicutes and Bacteroidetes, which typically represent up to 80% or more of the microbiota, with smaller contributions of Actinobacteria (∼3%), Proteobacteria (∼1%), Verrucomicrobia, and Fusobacteria (∼0.1% or less) (Arumugam et al., 2011; Hakansson and Molin, 2011; King et al., 2019).

As noted above, most studies of the effect of radiation on the GI microbiome have been conducted in the context of cancer radiotherapy, and recent reviews summarize the literature in that context (Liu et al., 2021; Tonneau et al., 2021). Indeed, therapeutic abdominopelvic radiation exposure frequently results in intestinal dysfunction and dysbiosis, with acute radiation enteritis complications observed in 50% or more of abdominally irradiated cancer patients (Touchefeu et al., 2014). Radiation enteritis is associated with high morbidity and mortality, and chronic symptoms as severe as rectal hemorrhage, strictures, and fibrosis develop 3 months to 20 years after completion of radiotherapy (Packey and Ciorba, 2010; Ding et al., 2020). However, these studies can shed light on what may happen in the event of a radiological or nuclear mass casualty incident in which victims exposed to more than 6 Gy of radiation may acutely experience nausea, vomiting, diarrhea, sepsis, and death (Wojcik, 2002).

Rapidly dividing human cells are the most sensitive to the damaging and killing effects of ionizing radiation (Donnelly et al., 2010), and in particular, the GI epithelium is very sensitive to radiation, given that the GI crypt rapidly divides to shedding villi cells every 2–4 days (Novak et al., 1979; Somosy et al., 2002; Clevers, 2013; Williams et al., 2015). Radiation-induced cell death leads to loss of GI epithelial integrity and function, leading to inflammation and penetration of the GI epithelial barrier by the luminal contents and microbiota (François et al., 2013; Shukla et al., 2016). In addition, radiation damage to endothelial cells of the blood vessels within the villi can also result in vascular damage, causing further inflammation and sepsis (Paris et al., 2001). In the context of radiotherapy, most acute symptoms generally resolve within a few weeks as mucosal crypt, and villus structures are reconstituted from surviving stem cells (Umar, 2010).

A diverse and healthy commensal intestinal microbiota plays an essential role in GI homeostasis. However, it has been found that severe postirradiation enteropathy is associated with low mucosal bacterial diversity (Ferreira et al., 2019). In rodent studies, specific findings of microbiota changes in postirradiation fecal samples include increased abundance of the phylum Proteobacteria and family *Lactobacillaceae* and decreased abundance of families *Lachnospiraceae*, *Ruminococcaceae*, and *Clostridiaceae*, with some changes observed out to 10 months (Lam et al., 2012; Goudarzi et al., 2016; Zhao et al., 2019; Li et al., 2020). In humans, one prospective study of nine gynecologic cancer patients found that Firmicutes and Fusobacterium phyla were significantly decreased in fecal samples pre- versus post-pelvic irradiation (Nam et al., 2013). While there are few prospective studies that document changes in the gut microbiota postradiation, growing research interest in this area will likely fill that gap.

### Oral Microbiome

The microbiota in the oral cavity has long been studied, as changes in the balance of flora in the oral cavity can lead to infections like candidiasis, also known as “thrush,” first described and attributed to a fungus in 1839 by Bernhard von Langenbeck (Hellstein and Marek, 2019). Hundreds of years of interest and easy access to the oral cavity and saliva samples have facilitated extensive research on the oral microbiome and its connection to various disease processes, including responses to radiation exposure (Anjali et al., 2020; Belstrøm, 2020). In the oral cavity, there may be from 10^8^ to 10^10^ CFU per gram of saliva (Lazarevic et al., 2009). It should be noted that the oral microbiota even in healthy people varies drastically across location in the oral cavity, time of day, hydration, what and when the person ate, oral hygiene, age, smoking status, and so on (Aas et al., 2005; Bik et al., 2010; Cameron et al., 2015; Leake et al., 2016; Hall et al., 2017; Belstrøm, 2020; D’Angiolella et al., 2020). In radiation exposures, oral side effects such as xerostomia (dry mouth) are seen in patients receiving external beam radiotherapy to the head and neck (Wijers et al., 2002; Dirix et al., 2006) and radioiodine therapy (Alexander et al., 1998; Solans et al., 2001; Jeong et al., 2013; Hollingsworth et al., 2016). In fact, in a follow-up Chernobyl study, 4 of 15 survivors reported experiencing xerostomia (Gottlöber et al., 2001). Salivary damage and subsequent dry mouth can lead to a variety of problems, from difficulty chewing and talking to increased dental caries, oral mucositis, osteonecrosis, and so on (Dirix et al., 2006; Gomez et al., 2011; Tolentino et al., 2011; Sroussi et al., 2017; Chen et al., 2020). While studies of the oral microbiome following a nuclear accident are limited, there are many research studies that examine the changes in the oral microbiota following head and neck radiation exposure in oncology (Anjali et al., 2020).

The oral cavity has a delicate microbiota balance that can be directly affected not only by irradiation but also from changes in saliva composition and/or volume due to radiation-induced damage of the salivary glands, which are particularly radio-sensitive organs (Kałużny et al., 2014). Since the 1970s, radiation-induced xerostomia has been known to affect the oral microbiota (Brown et al., 1975; Brown et al., 1978; Sroussi et al., 2017; Mougeot et al., 2019; Breslin and Taylor, 2020), and it has been recently discovered that *Candida* infections in patients who received radiotherapy are often from species that are more virulent and drug-resistant (Tarapan et al., 2019). This is particularly concerning, given that *Candida* is the fourth most common cause of bloodstream infections among hospital patients in the United States and can be fatal (Hajjeh et al., 2004; Lone and Ahmad, 2019). A number of studies found increased abundance of Gram-negative and *Lactobacillus* bacterial species, as well as *Candida* fungal species following radiotherapy (Vanhoecke et al., 2015). Indeed, Nishii et al. found oral candidiasis occurred in 31% of 326 oral/oropharyngeal cancer patients who underwent radiotherapy, with oral mucositis associated with a higher incidence of oral candidiasis (Nishii et al., 2020). Researchers collected buccal swabs from oral cancer patients before and after radiotherapy, and while these patients already had altered oral microbiota with high prevalence of certain species following radiotherapy such as *Streptococcus* pathogenic *Candida albicans*, *Klebsiella*, and *Pediococcus*, with elevated *Candida* and *Pediococcus* persisting out to 6 months (Anjali et al., 2020).

Another study found *Streptococcus* and other species were predictive of high-grade oral mucositis, while *Lactobacillus* and *Staphylococcus* were only detected in patients with low- or no-grade oral mucositis in a study of 19 patients receiving fractionated radiotherapy (Vesty et al., 2020). Patients who developed more severe oral mucositis following radiotherapy had a higher abundance of *Actinobacillus* (Zhu et al., 2017), and an increase in certain microbes that coincided with the onset of severe mucositis over the course of patients’ radiation treatment (Hou et al., 2018). Additionally, an *in vitro* study found ionizing radiation increased the adherence of *Streptococcus mutans* on dental restoration material and promoted the formation of biofilms (Cruz et al., 2010).

In addition to the risk of salivary and oral damage caused by prompt exposure during a radiation incident, radioactive iodine fallout can find its way into the environment and eventually into human bodies, leading to a well-documented increased risk in thyroid cancer (Robbins and Schneider, 2000; Cardis and Hatch, 2011; Thomas, 2018). Salivary glands (La Perle et al., 2013) express the sodium iodide symporter, facilitating radioiodine uptake and potential damage. Although little research on the impact of radioiodine on the oral microbiome has been conducted, given the similarities in damage and symptoms between radioiodine therapy and external beam radiotherapy, changes to the microbiota may be similar.

### Skin Microbiome

With a surface area of approximately 2 m^2^, the skin is the largest organ and is highly complex, with structures such as hair follicles and sweat ducts increasing its true surface area to about 25 m^2^ (Gallo, 2017). The variable surface of the skin supports a vast ecosystem of distinct microorganisms, where more exposed areas tend to be drier and less populated by resident bacteria (Roth and James, 1988). However, the overall number of microorganisms present on the skin is held relatively constant under normal conditions (Davis, 1996). The commensal relationship between cutaneous tissue and the diverse community of microorganisms plays a critical role in barrier protection from invading pathogenic microorganisms, homeostasis, and the adaptive immune response (Dréno et al., 2016; Sfriso et al., 2020).

Much is still to be learned of the interplay between the skin microbiome and ionizing radiation-induced cutaneous injury. Most clinical studies focus on posttreatment inflammation, particularly dermatitis in breast cancer patients after radiotherapy (Eslami et al., 2020). As it is very likely that many individuals will have cutaneous and combined injuries following a radiation mass-casualty incident, mediating changes in the skin microbiota with preventative or mitigative treatments is of particular importance for chronic and acute wound healing outcomes and to prevent systemic complications. Combined injury, consisting of total body irradiation (TBI) followed by punch wounding resulted in early detection of bacteria in the blood, heart, and liver, although detection of bacteria was delayed in mice that received radiation alone. Only transient bacteremia occurred in mice that underwent wounding alone. Results suggest that increased levels of iNOS, cytokines, and bacterial infection triggered by combined injury may contribute to mortality in this model (Kiang et al., 2010).

Thermal and radiation burns are also likely during a radiation incident. However, standard medical management for thermal burns such as medications, wound dressings, therapy, and surgery may not be appropriate for radiation burns, which have a different damage profile with cyclic waves of inflammation and progressive lesion formation over time (DiCarlo et al., 2020). Adding to this complex scenario is the possibility of bacterial infection. Researchers have demonstrated extremophilic bacteria such as *Aeribacillus*, likely introduced during debridement of flame or scald wounds, correlated with patient comorbidities, such as pneumonia, infection, and sepsis (Plichta et al., 2017). Germ-free mice have been shown to have accelerated wound closure and scar reduction with elevated levels of anti-inflammatory cytokine IL-10, angiogenic growth factor VEGF, and angiogenesis in the germ-free wound tissue, suggesting the influence of an inflammatory component in wound healing (Canesso et al., 2014). A few case reports of mesenchymal stem cell treatment of patients with severe radiation burns also showed a resolution of inflammation (Bey et al., 2007; Bey et al., 2010). Although these studies suggest bacteria delay skin injury healing, certain bacterial species, such as *Lactobacillus plantarum*, can inhibit biofilm growth of harmful bacterial (e.g., *Pseudomonas aeruginosa*), subsequently improving tissue repair (Valdéz et al., 2005). These studies suggest it is possible to harness the beneficial power of the skin microbiome, expanding therapeutic options.

Although different from radiation injury, the microbiome research conducted for other skin injuries, such as those involving ultraviolet irradiation (Wolf et al., 2016; Patra et al., 2019; Patra et al., 2020), diabetic ulcers, and other chronic skin diseases (Wolcott et al., 2016; Johnson et al., 2018), may shed light and help guide future skin microbiome research in the context of radiation injury. Additionally, clinical strategies currently used to treat these complicated skin wounds may provide insight into identifying effective therapeutics and improving patient outcomes. While a wealth of information can be found in the literature on processes governing wound healing, the role of the skin microbiome is less clear. Research shows that differences exist between normal and pathological microbial responses after a skin injury (Singer and Clark, 1999; Schultz et al., 2011; Johnson et al., 2018); therefore, a better understanding of the skin microbiome and its influence on the immune response has great medicinal potential with regard to radiation injuries.

### Lung Microbiome

Historically, lungs have been considered sterile. When it was first reported in 2010 that the microbiome in the lower airways was comparable to the upper bowel, the phenomenon was attributed to possible contamination during the bronchoalveolar lavage (BAL) procedure (Hilty et al., 2010). Since then, the existence of a microbiome in healthy lung has been widely accepted (Kiley and Caler, 2014; Mathieu et al., 2018). The lung microbiome is situated in the lower airways of healthy lung and houses a large number of microbes, including phyla Bacteroidetes and Firmicutes (Charlson et al., 2011; Dickson et al., 2013; Liu et al., 2020). The microbiome landscape changes dramatically under disease conditions affecting the lung, such as asthma and chronic obstructive pulmonary disease (Segal et al., 2014; Evsyutina et al., 2017), through processes involving immigration, elimination, and local growth conditions (Evsyutina et al., 2017).

Microbial migration occurs via air inhalation, micro-aspiration, and direct dispersion through the respiratory tract mucosa, while microbiome elimination occurs by mucociliary clearance, cough, and immune mechanisms. Microbiome growth conditions can be influenced by pO_2_, pH, blood perfusion, alveolar ventilation, temperature, lung epithelium, mucociliary clearance, and inflammatory cell activity. Furthermore, microbiome expansion is affected by bacteriostatic activity from surfactant produced in the distal alveoli. Finally, under disease conditions, the lung microbiome can be entirely destroyed and replaced with a single pathogen, as can occur during pneumonia (Araghi, 2020). Interestingly, the gut microbiota can affect general pulmonary health through a vital cross-talk between the gut microbiota and the lungs, referred to as the “gut–lung axis” (Keely et al., 2012). The gut–lung axis is bidirectional, denoting that the endotoxins and microbial metabolites released into systemic circulation by the gut can affect the lung, and if inflammation occurs in the pulmonary tissue, the gut microbiota is also affected (Dumas et al., 2018).

Though progress has been made, lung microbiome research is complicated by the difficulty in collecting biospecimens specific to the lung and lower airways. Clinically, sputum is used as a surrogate for lower airway samples; however, this process leads to contamination from microbes inhabiting the upper airways and oral cavity. Unfortunately, other than sputum, there are few reliable approaches to lower airway sampling, which is an obstacle to large-scale investigations of lung disease for studies requiring frequent sampling. Similarly, lung microbiome analysis using BAL fluid can also be contaminated by contributions from upper airway microbiota. Several studies analyzing lung tissue acquired via sterile surgical explant demonstrated that the lower respiratory tract contains a microbiome that is distinct from but related to that of the upper airways (Dickson et al., 2013).

While there are some publications related to radiotherapy and lung microbiome, there are no publications specific to the role of lung microbiome in radiation-induced lung injury at the writing of this review. One study described the prophylactic (pre-irradiation) use of heat-inactivated *Salmonella typhimurium* in ameliorating thoracic radiation-induced lung injury in mice by reducing apoptosis, inflammation, and endothelial mesenchymal remodeling of lung tissue (Kun et al., 2019). Some recent publications indicate that low-dose radiation therapy can be used in treating SARS-CoV-2–induced pneumopathy (Prasanna et al., 2020; Salomaa et al., 2020; Wilson et al., 2020); however, the relationship to the normal lung microbiome and the potential for a mitigation or biodosimetry strategy from these few studies is relatively unclear. Researchers in the radiation community can draw upon publications on the microbiome of the lung to better understand the significance of the microbiome in radiation-induced lung injury and how the microbiota are implicated in intervention strategies. These could include determining (1) whether an altered lung microbiome initiates radiation-induced disease pathogenesis, promotes chronic inflammation, or is merely a marker of injury and inflammation; (2) whether the lung microbiome can be manipulated therapeutically to change radiation-induced lung disease progression; and (3) what molecules (metabolites) generated during an inflammatory response can serve as biomarkers for pulmonary injury diagnosis and prognosis of the therapeutic interventions.

### Other Microbiota Niches

The following microbiome niches are of lesser interest to the radiation emergency mission space. Radiation damage to these systems has low to no impact on lethality and no well-established animal models of injury. However, radiation exposure can still greatly damage these tissues and their resident microbiota and have been included here for completeness.

#### Nasopharyngeal Microbiome

Contrary to the lung, the nasopharyngeal and upper respiratory tracts are more accessible, making their microbiota easier to study. Predominant bacterial phyla in the healthy nares include Actinobacteria and Firmicutes (Frank et al., 2010). In addition, postirradiation rhinosinusitis is a well-documented side effect of radiotherapy of the nasopharyngeal, sino-nasal, or skull areas, occurring in up to 45% of patients (Huang et al., 2007; Su et al., 2014; Maxfield et al., 2017). Chronic rhinosinusitis has long been characterized by sinus microbiome dysbiosis (Cope et al., 2017), but only more recently have microbiota changes associated with chronic rhinosinusitis following radiotherapy been studied. Temporal changes in the nasopharyngeal microbiota following radiation therapy were noted in 39 nasopharyngeal carcinoma patients, which were followed for 3 months after radiation therapy (Huang et al., 2021); however, these changes were similar to findings reported in unirradiated patients with chronic rhinosinusitis (Abreu et al., 2012). Furthermore, evaluation of sino-nasal swabs of 22 patients with chronic rhinosinusitis at an average 1.5 years after radiotherapy showed cultures dominated by many unique phyla of bacteria (Stoddard et al., 2019), which were similar to species found in unirradiated individuals with rhinosinusitis (Cope et al., 2017). This suggests that radiation can cause chronic rhinosinusitis, but the dysbiosis found is not distinct from chronic rhinosinusitis from other causes.

#### Urogenital Microbiome

Like the lung, the urinary tract and bladder were long thought to be a sterile environment, unless in a disease state. Only recently has more extensive research into the microbiome of the urologic system been conducted. Difficulties involved in obtaining bladder tissue samples from healthy individuals explain why its microbiome has yet to be extensively studied. A review of research done in this area discusses microbiota studies of urine and seminal fluid from prostate cancer patients, although changes in the urinary tract microbiota in response to radiation have yet to be explored (Aragón et al., 2018).

The vaginal microbiota, on the contrary, has been studied for over a century in the context of postmenopausal changes, with evidence emerging that *Lactobacillus* species dominate the microbiota and are vital for microbiota homeostasis (Ravel et al., 2011; Muhleisen and Herbst-Kralovetz, 2016; Buchta, 2018). Unlike the microbial diversity found in the healthy GI tract, the healthy vaginal microbiome is not normally phyla diverse, and an increase in bacterial diversity is an indication of vaginal dysbiosis (Muhleisen and Herbst-Kralovetz, 2016; Buchta, 2018). Indeed, one study found higher bacterial diversity in the vaginal microbiota following radiation in gynecologic cancer patients, who already had decreased lactobacilli abundance and increased diversity compared to healthy patients prior to radiotherapy (Tsementzi et al., 2020). Lactobacilli utilize glycogen and produce lactic acid which acidifies the vagina, protecting it from some infections (Buchta, 2018). Additionally, some species of lactobacilli appear to distinguish idiopathic infertile women from fertile women, indicating the vaginal microbiota is inextricably linked to reproductive health (Campisciano et al., 2017). Furthermore, low abundance of any *Lactobacillus* species has been linked to vulvovaginal atrophy which may put individuals at a higher risk of infection (Brotman et al., 2014). Changes to the vaginal microbiota have been studied in patients who received radiotherapy, which can sometimes induce menopause and subsequently decrease vaginal lubrication. Similar to the oral cavity, this change in environment alters the makeup of the microbiota and can lead to sexual and urinary organ problems, such as recurrent urinary tract infections (Portman and Gass, 2014). Specific taxa have been found to increase in abundance in the vaginal microbiota post- vs. pre-radiotherapy for gynecologic cancers including the family *Lachnospiraceae* (Tsementzi et al., 2020) and genera *Mobiluncus*, *Atopobium*, and *Prevotella* (Bai et al., 2019). Interestingly, an increase in cervical bacteria has been noted, with no difference in proportions, when culturing cervical swabs taken before and after external beam radiotherapy, suggesting the method of bacterial analysis and the location of samples affect the results (Mubangizi et al., 2014). These results suggest the microbiome may be involved in the mild reproductive and fertility effects seen in Chernobyl incident survivors (Cwikel et al., 2020) and nuclear industry workers (Doyle et al., 2001).

#### Ocular/Lacrimal Microbiome

The microbiota on the ocular surface, in tears and conjunctival fluid, and in lacrimal glands and ducts is only beginning to be considered. Studies among healthy patients found the genera *Corynebacterium* and *Pseudomonas* dominated the ocular microbiome (Huang et al., 2016; Suzuki et al., 2020). Studies of diseased state microbiota have been conducted in patients with dry eyes (Willis et al., 2020; Andersson et al., 2021), obstruction (Curragh et al., 2020), and Sjogren’s syndrome (Trujillo-Vargas et al., 2020). Although dry eyes are a known side effect of radiotherapy (Nuzzi et al., 2020) and radioiodine treatments (da Fonseca et al., 2016), research in the area of radiation impact on the lacrimal or ocular microbiota has yet to be conducted.

Research on the microbiome, including interactions with other microbiota across the body and their human host, is ever expanding. Studies of the impact of acute radiation exposure on many areas of the microbiome are still needed, although some studies may be difficult due to access challenges, and differences between animal and human microbiomes.

## Animal Models of Radiation Effects on Microbiome

Researchers have used standard TBI or partial-body irradiation (PBI) models to study the effects of irradiation on the microbiome, and the influence of the microbiome on radiation injury. Rodent models are especially useful because researchers can build on the vast literature in rodent radiation models, and many research tools are available. These studies tend to focus on the gut microbiome and its complex interplay with the immune system.

One challenge in earlier studies that examined the effects of irradiation on acute intestinal injury (GI-ARS) is that levels of radiation necessary to cause lethal GI-ARS caused significant death from just the hematopoietic syndrome of the acute radiation syndrome (H-ARS). Although myeloablation can be ameliorated by bone marrow transplant or compensated by only looking at an earlier survival time point, more recent rodent models have employed partial body shielding, which spares enough bone marrow to allow the immune system to provide some level of protection against infection and hemorrhage, and to accelerate immune reconstitution (Booth et al., 2012; Fish et al., 2016). Shielding of 5% (or lower) of bone marrow is thought to simulate the level of shielding that would occur during an actual large-scale nuclear exposure because people will likely be indoors and thus partially shielded (Booth et al., 2012). On the contrary, localized irradiation or higher levels of shielding may be closer to the clinical experience. The various models used, and what has been learned from them are described below.

The role of infection due to bacterial translocation from the gut has long been a recognized consequence of ionizing radiation in mammals; therefore, a series of studies using mice that have no gut flora (derived and raised in germ-free environment) from the Notre Dame Lobund Laboratory’s germ-free mouse colony were performed. In an initial study in mice, germ-free and conventionally housed mice were exposed to a range of radiation exposures of between 5 and 30 Gy (Wilson, 1963). In the radiation range corresponding to the hematopoietic syndrome (6–7 Gy), 30-day survival was higher in the germ-free animals. For higher radiation exposures, where all mice are expected to be dead by day 30, germ-free mice had a longer mean survival time (MST). These observations were confirmed in germ-free and conventionally housed mice as well as germ-free mice fed *E. coli* to populate the gut (McLaughlin et al., 1964).

In two subsequent articles, the MSTs and pathologies in mice receiving a range of radiation exposures were compared and described. Matsuzawa described four phases of radiation injury as radiation exposure was increased, corresponding to hematopoietic, heme/GI, GI, and CNS syndromes (Matsuzawa, 1965). Only in the last phase was no difference found in MST. Matsuzawa noted that the major difference in pathologies observed was increased septicemia in mice from the conventionally housed heme/GI group and later appearance of diarrhea in the GI group. This delay in the appearance of intestinal lesions was also observed for neutron-gamma mixed-field irradiation (Jervis et al., 1971). Further histopathological analysis of mice irradiated with 30 Gy showed differences in the epithelial cell counts of the intestinal crypts and villi, with irradiated conventionally housed mice having lower cell counts than their germ-free counterparts (Matsuzawa and Wilson, 1965).

From these studies, we can conclude that the microbiome has an influence on disease progression following radiation exposure; however, it was not until later that researchers elucidated which bacterial groups could have positive or negative influences on survival. It was found, for example, that the survival of germ-free mice reconstituted with normal human fecal bacteria had reduced survival when irradiated with 6.5 Gy compared to mice reconstituted with facultative anaerobic bacteria (Hazenberg et al., 1981). Around the same time, Onoue *et al.* found that the types of bacteria introduced into germ-free mice influenced the survival (diminishing with *Escherichia*, *Streptococcus*, *Pseudomonas*, and *Fusobacterium* or improving with *Clostridium*, *Lactobacillus*, or *Bifidobacterium* genera) when mice were exposed to 20 Gy of radiation (Onoue et al., 1981).

A subsequent study which directly examined the role of the microbiome in radiation injury also noted in a TBI model that germ-free animals were more radioresistant than those conventionally raised (Crawford and Gordon, 2005). In this study, mice were exposed to 16 Gy of radiation and given bone marrow transplants to allow them to survive H-ARS. Colonization of germ-free mice with *Bacteroides thetaiotaomicron* (obligate anaerobe) and/or *E. coli* (facultative anaerobe) prior to irradiation did not affect the relative radio-resistance of the germ-free mice, indicating that these species were not responsible for the radiation sensitivity of the mice with normal gut flora. In another study, mouse models of both TBI and fractionated total abdominal irradiation (TAI), in which 8 fractions of 4 Gy radiation was delivered to the mouse abdomen, were examined (Riehl et al., 2019). Pre-irradiation administration of lipoteichoic acid was found to protect mice given 7 or 8 fractions of radiation by 50%. Others utilized a localized rectal irradiation mouse model, which simulates pelvic radiation therapy provided in the clinic, finding a disruption in the colonic microbiome accompanied by an increase in TNFα, IL-1β, and IL-6 in the irradiated mice. These results suggest that radiation-induced disruption of the gut flora increases levels of pro-inflammatory cytokines (Gerassy-Vainberg et al., 2018). In other experiments utilizing a TBI mouse model (8.0–9.2 Gy), the role of the microbiome of “elite survivor” mice and its radioprotective effects were explored (Guo et al., 2020). This study is discussed in more detail below.

Although these models that provide information on the interplay between the gut microbiome and the immune system may mimic clinically relevant radiation exposures, they are not aligned with models currently being used to test radiation MCMs. Focal or organ-based radiation exposures do not simulate the expected situation in a mass casualty event, in which outcomes would be based on most if not all tissues being exposed to high radiation doses. Currently accepted irradiated animal models use shielding of ∼2.5–5% of the bone marrow as discussed above, which provides sufficient sparing to allow for survival past the H-ARS phase (Booth et al., 2012; MacVittie et al., 2019). Therefore, studies using these relevant animal models are needed to better understand the potential impact of the microbiome in radiation exposures similar to those expected during a public health emergency.

The gut microbiome has also been studied indirectly in animal models of radiation injury by testing various antibiotic regimens. The choice of antibiotics in these rodent studies has been influenced by clinical practice and recommendations for patients from groups such as the Infectious Diseases Society of America (IDSA) (Freifeld et al., 2011). Radiation exposure leads to bone marrow myelosuppression, and the neutropenic patient is susceptible to bacteremia from gut bacteria translocation (Waselenko et al., 2004). Therefore, studies were carried out to determine if mitigation of neutropenia can affect survival and other outcomes in animal models subjected to lethal doses of radiation (Plett et al., 2012; Farese et al., 2013; Chua et al., 2014; Hankey et al., 2015; Zhong et al., 2020). These experiments showed that administration of granulocyte (G)– or granulocyte–macrophage (GM)–colony-stimulating factor (CSF) rescued animals from H-ARS and reduced bacteremia in the nonhuman primate (NHP). The use of antibiotics in treatment of radiation exposure is further discussed below.

While mouse models are frequently studied to determine involvement of the microbiome in radiation exposure outcomes, other models have been adapted to explore the relationship between radiation and the microbiome. For example, a TBI rat model (employing single or fractionated radiation exposures) has been used to examine changes in 16S rRNA gene sequences from fecal samples (Lam et al., 2012). Although the goal was to develop a predictive biomarker for gut radiation exposure, the pattern of changes in the microbiome could not be compared to radiation-induced microbiome changes in other animal model species. Even germ-free mice that have undergone fecal microbiota transplantation (FMT) with human microbiota do not fully recapitulate the physiological human microbiota and microbiome, likely due to species microenvironmental differences (Turnbaugh et al., 2009; Nguyen et al., 2015). FMT is discussed in more detail below.

A number of large animal models of H- and GI-ARS have been developed to improve the understanding of the natural history of radiation injuries. These models include NHPs, typically Chinese rhesus macaques (*Macaca mulatta*), and Göttingen minipigs (*Sus scrofa domestica*). These models have been developed as preclinical models to more closely represent human anatomy, tissue structures, and physiology, and to predict human responses to radiation (MacVittie et al., 2012; MacVittie et al., 2012; Elliott et al., 2014). For example, researchers have examined microbiome changes following TBI in both of these larger animals (Carbonero et al., 2018; Carbonero et al., 2018). These studies suggest that the minipig microbiota may more closely reflect that of humans, with a similar distribution and response to radiation exposure. Examining 16S rRNA from pre- and postirradiation fecal samples revealed that some bacterial species normally found intracellularly, and not in the colonic lumen, were increased in postirradiation fecal samples in both minipigs and mice. Although there were some similarities in the microbiome profiles among the mouse, rhesus macaque and minipig models (Goudarzi et al., 2016; Casero et al., 2017; Carbonero et al., 2018; Carbonero et al., 2018; Gerassy-Vainberg et al., 2018), there were also notable differences. Therefore, application to the human experience should be approached with caution. In addition, the minipig model uses a higher level of shielding (55%) that would not necessarily be as applicable to a mass casualty situation (Measey et al., 2018; Measey et al., 2018). Also noteworthy is that animal care procedures can influence these results. For example, NHPs included in these studies received antibiotics for 3 days after irradiation, potentially confounding the microbiome results. These inter-species comparisons reinforce that for these animal models to be useful, they must ultimately be linked to the growing knowledge of the human microbiome and the effects of irradiation on people. Additionally, it is important to note that the nature of animal models including closely related strains of species and “well-housed environments” affect the microbiome in ways not reflective of real-world scenarios.

## The Effects of the Microbiome on the Radiation Response

The delicate balance between the host and its microbiota can affect patient outcomes in the areas of cancer (Liu et al., 2019), immuno- (Sivan et al., 2015; Tanoue et al., 2019), and radio-therapy (Roy and Trinchieri, 2017), as well as colorectal surgery (Chen et al., 2018). The host–microbiota interaction is a symbiotic one that needs careful consideration as potential MCMs are proposed to modulate the microbiota. Consequently, approaches such as antibiotics, probiotics, dietary modifications (including prebiotics, vitamins, and minerals), and fecal microbiota transplant could represent treatments that may alter survival outcomes after radiation exposure (Table 2). Additionally, changes in the microbiota could be used as biomarkers to indicate the severity of radiation injury and/or the efficacy of treatments. Below are targeted treatments that modulate the microbiome and in turn minimize radiation injuries.

**TABLE 2:** Targeted treatments that modulate the microbiome and radiation response.

Antibiotics	Doxycycline (Plett et al., 2012)
	Neomycin (Plett et al., 2012)
	Enrofloxacin (Waselenko et al., 2004)
	Tetracycline (Waselenko et al., 2004)
	Ciprofloxacin (Plett et al., 2012)
Probiotics	*Lactobacillus rhamnosus* GG (LGG; Culturelle®) (Dong et al., 1987)
	*Bifidobacterium longum* (Khailova et al., 2013)
	*Lachnospiraceae *(Guo et al., 2020)
	*Enterococcaceae* (Guo et al., 2020)
	*Lactobacillus reuteri*-producing IL-22 (Zhang et al., 2020)
Diet	Prebiotics: non-digestible dietary fibers (e.g., apple pectin) (Garcia-Peris et al., 2016; Yang et al., 2017)
	Hydrogen-water (Xiao et al., 2018)
	Omega-3 polyunsaturated fatty acids (Zhang et al., 2019)
	Vanillin (Li et al., 2019)
	Vitamins D, E, and C (Huang et al., 2019; Segers et al., 2019)
	Flavonoids (Turner et al., 2002)
	Polyphenols (Turner et al., 2002)
	Folic acid (Turner et al., 2002)
Fecal Microbiota Transplant	Short-chained fatty acids (Li et al., 2020; Xiao et al., 2020)
	Indole 3-propionic acid (Li et al., 2020; Xiao et al., 2020)
Others	4-Nitro-phenyl-piperazine pharmacophore (Micewicz et al., 2019)
	Phycocyanin (Lu et al., 2019)

### Antibiotics

Similar to H-ARS, chemotherapy can induce myelosuppression in cancer patients, resulting in increased risk of infection. Thus, the IDSA has published guidelines recommending neutropenic cancer patients be given fluroquinolone antibiotics (Freifeld et al., 2011). As it is likely that antibiotics will be first-line therapeutics in the event of a mass casualty radiation emergency (Coleman et al., 2015), this IDSA recommendation was initially put forward as a recommendation of the Strategic National Stockpile Radiation Working Group, convened in 2002 (Waselenko et al., 2004). This guidance is supported by studies carried out in mice at various institutions. For example, in developing a model of H-ARS, investigators tested several antibiotic regimens in mice given various doses of TBI—finding MST was increased in antibiotic-treated mice, although levofloxacin did not provide a better outcome than ciprofloxacin (Plett et al., 2012). They also found that the use of different combinations of antibiotics (e.g., doxycycline + neomycin) increased survival (Plett et al., 2012).

Additionally, iliac bacteria counts in mice exposed to 10 Gy of TBI were found to be reduced, and anaerobe repopulation was delayed (Brook et al., 1988). Anaerobic bacteria appear to be protective, as treatment with metronidazole caused a further decrease in the anaerobic population and quicker onset of mortality. A subsequent review (Brook et al., 2004) noted that administration of quinolones to mice reduced levels of Gram-negative aerobes while sparing the anaerobic population, which is in alignment with IDSA guidelines and is the preferred choice.

Researchers have long known that administration of antibiotics to irradiated animals can affect their survival, as noted above. This modification has generally been attributed to the ability of these molecules to reduce the likelihood of opportunistic infections in animals that are immunosuppressed—but what if the efficacy could also involve a more direct modification of the natural flora of the animal? Fluoroquinolones, such as enrofloxacin and tetracycline, have been shown to reduce radiation damage to hematopoietic progenitor cells grown in culture. Thus, the radiation dose-modifying effect of some antibiotics may allow them to serve as radiation mitigators in addition to their ability to slow the growth of microbes (Epperly et al., 2010). These findings were further explored in another model of GI-ARS that demonstrated that oral fluoroquinolones also led to higher survival rates in irradiated mice (Booth et al., 2012). In a mouse model of radiation combined injury, ciprofloxacin provided similar protection (Kiang et al., 2014), and in a TAI model, where radiation exposure was used to reduce the number of GI microbes, a cocktail of antibiotics given prior to radiation exposure improved bacterial regrowth in the gut (Zhao et al., 2020).

In addition, the use of acidified water, which is frequently employed in animal colonies, could mask the impact of radiation-induced GI injury. Acid water (pH 2.5–3.0) is used to prevent bacterial infections from spreading within an animal colony.^1^ It is often accomplished using hydrochloric or sulfuric acid or tetracycline (Hermann et al., 1982). Its use provides protection not only primarily against *Pseudomonas aeruginosa* but also against other Gram-negative organisms (Small and Deitrich, 2007), and in mouse models, water acidification has been shown to reduce the diversity of the gut microbiome (Sofi et al., 2014). Therefore, researchers considering the use of radiation injury models to study microbiome traits should be aware of these kinds of husbandry details in their animal facilities.

### Probiotics

The idea of altering the host microbiome was first introduced by Russian embryologist Elie Metchnikoff in the early 1900s (Podolsky, 2012). In the 1990s, a resurgence of probiotic research occurred and only in 2001 was the term “microbiome” used in the literature to describe the collective genome in a host. In late 2001, the Food and Agriculture Organization of the United Nations and the World Health Organization held an expert consultation in Cordoba, Argentina, to evaluate the health and nutritional properties of probiotics in food, which led to a joint report to provide assessment and safety guidelines for research in the field (Food and Agriculture Organization of the United Nations World Health Organization, 2006). Since then, many studies have demonstrated the beneficial effect that live, naturally occurring microorganisms can have on the immune system (Hardy et al., 2013; Peters et al., 2019), gut (Gourbeyre et al., 2011; Quigley, 2012), food allergies (Di Costanzo et al., 2020), colon (Pujo et al., 2020; Wang et al., 2020), skin (Friedrich et al., 2017; Patra et al., 2020), and central nervous system (Kim et al., 2020; Loniewski et al., 2020). Of particular importance for this review are the therapeutic effects of probiotics that are seen when these systems are exposed to ionizing radiation.

The Institut des Maladies de l’Appareil Digestif conducted a systematic review of six preclinical and seven clinical studies (Touchefeu et al., 2014), which found that decreases in *Bifidobacterium*, *Clostridium cluster XIVa, Faecalibacterium prausnitzii*, and increases in *Enterobacteriaceae* and *Bacteroides* after radiotherapy contributed to GI mucositis, leading to increased diarrhea and bacteremia. Many probiotic strains were investigated as preventative therapeutics, most of which led to a reduction in diarrhea or bacteremia incidence. Another systematic review considered 15 clinical trials studying varied GI pathologies (Picó-Monllor and Mingot-Ascencao, 2019). They concluded that a combination of probiotics could reduce the incidence of mucositis in chemo- or radiotherapy-treated patients. Likewise, a meta-analysis of randomized controlled trials showed that supplementation with *Lactobacillus acidophilus* plus *Bifidobacterium bifidum* had a modest effect at preventing radiation-induced diarrhea after abdominal or pelvic radiotherapy (Liu et al., 2017). Clearly, probiotics within *Lactobacillus* and *Bifidobacterium* genera were found effective in many of the trials.

Nonpathogenic bacterial species in genera such as *Lactobacillus* and *Bifidobacterium* are commonly used and have demonstrated a wide range of health benefits (Hardy et al., 2013). Understanding the role these bacteria play in the processing and biotransformation of xenobiotics or foreign compounds (e.g., drugs and antibiotics) in the host gut can lead to personalized therapeutics to avoid or circumvent antibiotic resistance (Maurice et al., 2013). In the case of a mass casualty radiation emergency, antibiotics will likely be used as first-line therapeutics (Coleman et al., 2015). Therefore, understanding this interplay will be essential to selecting the proper antibiotics. It may also be possible to co-administer a probiotic that can manage the microbial variability of the human gut.

Research on the potential for probiotics to serve as radiation MCMs is limited; however, the prophylactic use of probiotics has been explored extensively. The knowledge gained about underlying mechanisms in these kinds of studies could lead to druggable pathways and aid in the development of MCMs, specifically to address GI-ARS. For example, death was delayed for mice fed *Lactobacillus rhamnosus* GG (LGG) prior to exposure to 14 Gy of TBI (Dong et al., 1987). LGG, the first bacterial strain to be patented in 1989, has since demonstrated benefit against GI issues (Dong et al., 1987; Ciorba et al., 2012; Capurso, 2019; Riehl et al., 2019), perhaps by altering the immune system (Capurso, 2019), and protecting intestinal epithelium (Riehl et al., 2019). In another study, LGG protected the intestinal epithelium in mice that were administered the probiotic or LGG-conditioned media by oral gavage, 3 days prior to 12-Gy TBI (Ciorba et al., 2012). Researchers showed that LGG administration prior to irradiation increased the number of regenerative crypt cells and reduced epithelial cell apoptosis. This effect was observed both for mice administered LGG and mice administered LGG-conditioned media. Moreover, a head-to-head comparison of commercially available probiotics demonstrated that Culturelle offered a similar level of radioprotection to that produced by live, cultured LGG; however, protection was not provided by another non-*Lactobacillus*, commercially available probiotic (*B infantis* 35624; Align) (Ciorba et al., 2012). Administration of probiotics (LGG and *Bifidobacterium longum*) has also been shown to improve survival in pediatric mice after the onset of sepsis resulting from a cecal ligation and puncture (Khailova et al., 2013). In addition, several probiotic species were shown to be effective at displacing dangerous enteropathogens (Candela et al., 2008). Together, these studies suggest that *Lactobacillus* may be the probiotic genus of choice for ameliorating radiation-induced GI injury.

*Lactobacillus* is a member of the Firmicutes phylum, and another recent study found elevated Firmicutes bacteria levels in irradiated mice were associated with a survival benefit. Mice exposed to 9.2-Gy TBI that had an abundance of bacteria in the *Lachnospiraceae*, and *Enterococcaceae* families present in their gut had a significant survival advantage or were considered “elite-survivors” (Guo et al., 2020). Upon exposing germ-free mice to “elite-survivor” dirty cages or FMT via oral administration of feces, specific pathogen-free mice had significantly higher rates of survival than non-FMT controls. To substantiate these findings in humans, researchers also looked at fecal samples from 21 leukemia patients undergoing TBI as a pre-hematopoietic stem cell transplant conditioning. Patients with higher levels of *Lachnospiraceae* and *Enterococcaceae* generally had shorter bouts of diarrhea, as well as increased levels of propionate and tryptophan metabolites (Guo et al., 2020).

Second-generation probiotics are also being developed to take advantage of the natural properties of these bacteria, using microbial-mediated delivery of drugs to target the gut. Researchers have engineered probiotics that produce IL-22 (Zhang et al., 2020), a cytokine with anti-inflammatory properties known to stabilize both intestinal Paneth cells and Lgr5+ intestinal stem cells (Zha et al., 2019). In this study, C57BL/6 mice were exposed to 9.25-Gy TBI and then treated with *Lactobacillus reuteri*–producing IL-22 strains postirradiation via oral gavage. A 30% improvement in survival was noted, as compared to animals dosed only with the IL-22 protein. Time of administration of the bacteria was also examined, and a survival advantage could be seen even when dosed at 72-h postirradiation, with the highest benefit seen at 24 h (85%) and 48 h (70%) postirradiation administration (Zhang et al., 2020).

Probiotics may be therapeutic in systems beyond the GI. Oral probiotics have been found to affect microbial communities and local inflammation within these axes as well as the vaginal microbiota (Petricevic et al., 2008), skin (Eslami et al., 2020), and more. Additionally, the emerging information in the area of microbiome/gut–brain axis opens up new opportunities for the development of effective treatments for CNS disorders. Changes in the gut microbiota postirradiation have been associated with psychoneurological symptoms in cancer patients (Bai et al., 2020). Psychobiotics (bacterially mediated biotherapeutics, which include probiotics, prebiotics, and synbiotics—a combination of probiotics and prebiotics) are currently being investigated for their potential in treating neurologic disorders. Psychobiotics can be delivered through supplements, functional foods, and dietary changes (Long-Smith et al., 2020).

As the field of probiotics has continued to mature, researchers have found that synbiotics may provide a superior outcome than either one alone, by providing an optimal GI environment to allow the probiotics to survive and colonize the gut (Markowiak and Śliżewska, 2017). Another important consideration is the risk associated with certain strains of probiotics such as the *Enterococcus* genus, which can acquire antibiotic resistance and become pathogenic. To date, no enterococcal probiotics have been approved for human use, leading the European Food Safety Authority to conclude that “*Enterococci* do not meet the standard for Qualified Presumption of Safety” (Wang et al., 2020). Given these data, along with studies showing their systemic effects (Valdéz et al., 2005; Petricevic et al., 2008; Keely et al., 2012), probiotics are a promising potential treatment for GI-ARS and other radiation injuries.

### Diet, Prebiotics, Vitamins, and Minerals

In considering the GI microbiome, dietary supplementation can play a major role in the composition of gut bacteria and impact of radiation exposure. For example, normal tissue injuries from administration of abdominal radiotherapy to treat gynecologic malignancies can sometimes evolve into chronic radiation enteritis. Therefore, a clinical trial (NCT01549782) was carried out to study the effect of consumption of certain prebiotics, in this case fiber and plant sugars, on stool consistency in postirradiation patients (Garcia-Peris et al., 2016). Some improvement was noted in the group that consumed the prebiotic diet (reduction in days of diarrhea), suggesting that these dietary changes could lead to improved quality of life for these patients. Although the causal role of modulating microbiome by supplements to improve radiation injury resulting from accidental exposure to large doses is not as widely published, supplements are reported to protect gamma-irradiated mice (Shimoi et al., 1994) and improve survival (Satyamitra et al., 2011; Obrador et al., 2020). However, there are conflicting reports that underscore the need for caution in the use of all supplements without supporting data. For instance, investigators reported that high-protein diet such as methionine-supplemented diet (MSD) is used to build muscle mass in patients undergoing chemo- and/or radiotherapy; however, when this diet was fed to CBA/CaJ mice exposed to 3–8.5 Gy of TBI, the mice developed acute radiation toxicity, even at sublethal doses of 3 Gy, and demonstrated higher mortality (Miousse et al., 2020). Another study reported that MSD increased GI toxicity in abdominal irradiated CBA/CaJ mice, with a concomitant shift in gut microbiome, reduction in microbiome diversity, and significant increase in pro-inflammatory genus *Bacteroides* (Ewing et al., 2021). In addition, omega-3 polyunsaturated fatty acids were shown to reduce intestinal inflammation following radiotherapy (Zhang et al., 2019), a finding that was attributed to its ability to reduce oxidative stress in the GI tract. Similarly, consideration of the diet of astronauts has been a major source of concern, since space flight involves exposure to cosmic radiation (Turner et al., 2002). By providing extra antioxidants to the diet, in the form of vitamins such as E and C, as well as flavonoids, polyphenols, and folic acid, it may be possible to modify the composition of gut bacteria and reduce the risks associated with radiation exposure. This could be applicable to a wide range of scenarios involving radiation exposure including during space missions.

#### Prebiotics

The microbiome can be altered by various factors, but nondigestible dietary fibers, which serve as a food source, and can greatly influence the expansion of certain bacteria (Villéger et al., 2019). By regulating the presence or absence of key prebiotics, the microbiota can be changed, and thus, the metabolites produced by specific bacterial strains can also be enhanced to promote a positive outcome for the irradiated host (Louis et al., 2014). The addition of prebiotics has been shown to change the microbial community in the GI tract of irradiated mice and reduce intestinal permeability, leading to a decrease in the expression of inflammatory and oxidative stress markers (Cani et al., 2009). Another study showed that apple pectin could protect the terminal ileum and ameliorate radiation-induced intestinal fibrosis in mice by increasing the levels of short‐chain fatty acids and altering the intestinal microbiota (Yang et al., 2017). Additionally, hydrogen-water has been associated with ameliorating radiation-induced GI toxicity by maintaining a healthier gut microbiota composition (Xiao et al., 2018). Omega-3 polyunsaturated fatty acids have been shown to reverse intestinal microbial dysbiosis by increasing beneficial bacteria such as *Lactobacillus* and *Bifidobacterium* genera after chemotherapy and radiotherapy (Zhang et al., 2019). Prebiotics offer a source of enrichment to the microbiome; thus, their use can help optimize the gut flora and thereby regulate immune function. Such dietary interventions have a demonstrated role in the control of the inflammatory response and can potentially serve as a way to regulate inflammation after exposure to ionizing radiation.

A plant compound derived from vanillin (VND3207), a flavoring agent, has also been shown to mitigate GI-ARS through its action on modifying the composition of the bacteria in the gut (Li et al., 2019). C57BL/6J mice were irradiated (9-Gy TBI) and treated orally with VND3207 either prior to or following exposure. Animals that were pretreated had the greatest improvement in survival, although those treated postirradiation also saw a statistically significant survival benefit. Researchers determined that the structures of the microbiome of the gut were modified by the radiation exposure, and treatment with VND3207 modified the relative quantities of different bacterial species back to the level of unirradiated mice.

#### Vitamins and Minerals

Vitamin D has received attention for its role in immunity and inflammation (Lucas et al., 2014) and can be considered a master regulator in the modulation of the host microbiome (Ghaly et al., 2019). It contains fat-soluble secosteriods, responsible for absorption of calcium, magnesium, phosphate, and other trace elements needed for healthy biological functions (Huang et al., 2019). Vitamin D has also been associated with the treatment of inflammatory bowel disease (Fletcher et al., 2019), colorectal cancer (Abrahamsson et al., 2019), radiation dermatitis (Nasser et al., 2017), and pelvic radiotherapy (Castro-Eguiluz et al., 2018). Approximately 60% of radiotherapy patients receive vitamin D supplementation, as it is thought to enhance radiation resistance of healthy tissues by multiple mechanisms that reduce tissue inflammation and help with intestinal barrier function, by way of the microbiota (Huang et al., 2019). Studies with vitamin D–deficient mice showed a depletion of *Lactobacillus* and an enhancement of enteropathogens such as *Clostridium* and *Bacteroides* genera (Jin et al., 2015). In summary, vitamin D has been shown to play a key role in radiation resistance, but the underlying molecular mechanisms of its influence on the microbiome has yet to be completely elucidated. Some of these mechanistic pathways may be potential areas of exploration for MCM discovery.

It should be noted, however, that not all dietary approaches have proven to be successful in reducing the incidence of GI complications following anti-cancer radiotherapy. For example, a clinical trial that studied oral starch supplements to reduce radiation proctitis did not meet its primary endpoint in patients irradiated for cervical cancer (Sasidharan et al., 2019). Furthermore, in a mouse model of lethal radiation exposure, mice that received dietary supplementation with methionine were found to be more sensitive to GI-ARS (Miousse et al., 2020). Carried out in a PBI (hind leg shielded) model, investigators showed a change in the gut microbiome of the supplemented animals, which progressed to leakage, bacterial translocation, decreased citrulline levels, fewer crypts, and a reduced luminal surface area.

In a recent review, it was pointed out that clinical trials investigating the use of dietary modifications to mitigate the adverse effects associated with normal tissue injuries during radiation therapy involving the pelvis have yielded contradictory results (Segers et al., 2019). Approaches such as vitamins, pre- and probiotics, and a variety of food supplements have had varying degrees of success, leading the authors to conclude that clinical trial parameters involving reinforcing the gut microbiome with natural products should involve more definitive study endpoints and greater control of quality and optimization of dosing.

### Fecal Microbiota Transplant (FMT)

A novel investigative treatment is the use of FMT. Briefly, fecal material is obtained from a screened, healthy donor (or in the case of radiation exposure, an unirradiated host) followed by a dilution, homogenization, and filtration processing. The resulting preparation is then administered to the colon of the recipient, either through oral ingestion of a capsule, or via colonoscopy or enema. In preclinical studies, animals are typically fed donor feces. Initially conceived as a means of correcting the microbiome imbalance in individuals suffering from chronic GI infections, the therapy has completed a randomized, controlled clinical trial for treatment of antibiotic-resistant *Clostridium difficile* infection (Kelly et al., 2021). The therapy is believed to work by “out-competing” growth of *C. difficile* with other more protective species. Studies have shown that this treatment can mitigate infections in 80–90% of patients (van Nood et al., 2013). FMT procedures have also been studied to address a number of different disease states, such as multiple sclerosis (NCT03975413), diabetes (NCT04124211), autism (NCT03408886), AIDS (NCT02256592), and liver diseases (NCT03152188) (Lo, 2019). These findings of efficacy across multiple organ systems and disease states are not surprising, given the acknowledged role of the GI microbiome in the “gut–brain–skin axis” (Vojvodic et al., 2019) and the “gut–lung axis” (Dumas et al., 2018; Nie et al., 2020), which involve a close interplay between the systems and regulation by signaling molecules. Therefore, balance of microbes in the GI tract is important for maintenance of many conditions outside the gut.

#### Preclinical FMT Studies

Microbiome and FMT studies have been conducted in many animal models, including mice (Chen et al., 2020), rats (Yu et al., 2020), chickens (Metzler-Zebeli et al., 2019), pigs (McCormack et al., 2019), and NHPs (Hensley-McBain et al., 2016). There are many publications that document the potential for this unorthodox therapy (Wang et al., 2016; Cui et al., 2017; McIlroy et al., 2018; Villéger et al., 2019). For the purposes of this review, the focus will be only on its use for indications involving radiation.

The possible role of gut bacteria in the biological radiation response was suspected even as early as 1963, with the germ-free mice studies by Wilson (1963) and McLaughlin et al. (1964) discussed earlier. There have been several avenues of research that have specifically explored whether FMT could protect against high dose, TBI, or PBI exposures, which can lead to the development of the ARS. In one study, researchers noted that the composition of bacteria varied between male and female mice, a finding that correlated with the animal’s radiation sensitivity (Cui et al., 2017). When provided with FMT via oral gavage for 10 days using same-sex or opposite-sex donors, C57BL/6 mice exposed to 6.5-Gy TBI had increased survival, which was found to be highest when the donor sex matched the recipient. Function and continuity of the GI tract was also found to be improved in FMT-treated animals. Earlier studies by the same group had suggested that the known circadian rhythms affecting radiation sensitivity could also be linked to different bacteria present in the guts of animals subjected to altered light/dark cycles (Cui et al., 2016). In another study carried out in irradiated germ-free mice, fecal transfer from irradiated mice exhibiting radiation-induced dysbiosis to germ-free mice transmitted inflammatory susceptibility and increased susceptibility to GI radiation injury, which appeared mediated by IL-1β (Gerassy-Vainberg et al., 2018). As mentioned earlier, researchers showed that mice who received fecal engraftment from “elite survivor” mice had higher survival following TBI (Guo et al., 2020), further supporting the prospect of utilizing FMT as a MCM.

To exploit the many microbiota and functional changes observed with animal models in response to radiation, studies have been done to evaluate the usefulness of microbiota-derived short-chain fatty acids and other metabolic products as potential MCMs, to either prevent or mitigate radiation-induced GI injury. In a study in which FMT was given to irradiated mice, analysis of fecal pellets showed that a microbial molecule—indole 3-propionic acid (IPA)—was present at high levels (Xiao et al., 2020). Believing that this molecule could be responsible for the observed radiation protection obtained with FMT, oral IPA alone was provided to another group of irradiated animals. Treated animals had decreased inflammation and improved GI function after irradiation, suggesting its possible use as an effective MCM or radiotherapy treatment. Other studies found oral gavage of IPA and microbiota-derived valeric acid (VA) provided protection against up to 7 Gy, and, in the case of VA, mitigated GI radiation injury when given post-TAI (12 Gy). VA was found to prevent intestinal inflammation and dysfunction, and maintain microbiota compositional patterns (Li et al., 2020; Xiao et al., 2020).

The potential use of FMT has also been considered as a means of mitigating late effects attributable to prior radiation exposure, including in organ systems outside the GI tract. Given the “gut–lung axis” mentioned earlier, the GI microbiome is known to play a role in lung immunity; therefore, this finding has been explored as a potential treatment for pneumonitis in lung cancer patients treated with radiation (Nie et al., 2020). To study this, C57BL/6 mice were provided antibiotics prior to irradiation. In those animals, there was higher radiation mortality and more weight loss than in control animals. In addition, higher levels of lung damage were observed. When the same animals were then treated using FMT from untreated, unirradiated animals, lung inflammation and tissue damage were decreased, along with an alteration of the bacterial colonies found in the GI tract. The authors suggested that the tissue-type plasminogen activator might be involved in the inflammatory process.

#### Clinical FMT Studies

To date, there are more than 380 clinical trials^2^ involving FMT, many of which investigate FMT as a treatment for GI-targeted diseases such as *C. difficile* (Shogbesan et al., 2018), inflammatory bowel (Browne & Kelly, 2017), Crohn’s ulcerative colitis (Paramsothy et al., 2017; Blanchaert et al., 2019), chronic constipation (Ge et al., 2017), and radiation enteritis (NCT03516461). In the field of cancer and radiation oncology, radiation therapy to the pelvic or abdominal area is known to lead to GI damage in up to 50% of patients (Benson 3rd et al., 2004). A 2014 review explored the published literature for evidence that the GI tract microbiome played a role in this kind of damage (Touchefeu et al., 2014). Owing to these findings, clinicians began to consider the potential of FMT in radiotherapy, where a link was made between the microbiome of the GI tract and success of stem cell transplants for leukemia (Dougé et al., 2020). Results suggested that FMT could be used to rebalance the bacterial composition of the gut, and thereby reduce posttransplant complications. In addition, FMT has been proposed as a means of addressing chronic radiation enteritis, which has major quality-of-life implications. One trial (NCT03516461) of five female patients receiving pelvic radiotherapy found that FMT could mitigate serious chronic radiation enteritis-related complications such as diarrhea, bleeding, pain, and fecal soiling, and demonstrated the procedure to be safe (Ding et al., 2020). However, results suggest that caution should be employed when considering the use of FMT. For example, one case study described the use of FMT in a female patient who had received radiotherapy localized to the cervix (30 × 8 Gy) for treatment of a gynecologic cancer (Harsch and Konturek, 2019). The radiation treatment led to unpleasant GI complications that included diarrhea, malabsorption, and stenosis of the sigmoid portion of the colon, which she lived with for 17 years. When other therapies, including probiotics and dietary changes, did not provide relief, FMT was considered. Several days later after the transplant, the formation of a small bowel obstruction led to emergency surgery. Given the speed with which this complication arose after the FMT, clinicians speculated that the introduction of new species into the colon could have led to “trapping of a gut segment.” In summary, the use of FMT as a means of addressing radiation-induced injuries, not only to the GI tract but also to other organ systems, represents an intriguing possible treatment.

### Other Treatments for Radiation Injury Targeting the Microbiome

Novel therapeutics are being developed in search of effective MCMs against ARS, including radiation mitigators that have a common 4-nitro-phenyl-piperazine pharmacophore (NPSP) (Micewicz et al., 2019). In this study, C3H mice were exposed to an LD_70/30_ dose of radiation and then treated with an NPSP mitigator. To track long-term changes in the mice microbiota, fecal samples were collected from both irradiated and control mice on days 162, 214, and 442. The colonic microbiota was analyzed by 16S rDNA enrichment and sequencing, showing a consistent level of Firmicutes-to-Bacteroidetes composition in both treated and control mice until day 214. At this point, mice treated with NPSP 5355512 exhibited a decreased amount of Bacteroidetes, while the level of Firmicutes increased as compared to control mice (Micewicz et al., 2019). The Firmicutes-to-Bacteroidetes ratio is often analyzed as a marker for gut health but can fluctuate often and change with age (Mariat et al., 2009). While the significance of the change still needs to be elucidated, it is interesting to note that composition of the microbiome differed between the treated and non-treated groups.

Other therapeutics such as phycocyanin (PC), an active protein found in the genus *Arthrospira*, have been examined for efficacy against radiation-induced GI injury after radiotherapy. PC has been shown to have anti-inflammatory (Remirez et al., 2002) and antioxidant (Villegas et al., 2014) properties. In one study, C57BL/6 mice were administered PC daily for a month prior to an exposure of 12-Gy TAI (Lu et al., 2019). PC treatment provided protection against radiation-induced GI injury and maintained a healthier level of diversity in the microbiota, which is usually reduced after irradiation. In general, the levels of beneficial bacteria were increased, harmful bacteria were decreased, and inflammatory cytokines such as TNF-α and IL-6 were downregulated (Lu et al., 2019). Another drug simvastatin, commonly used to treat high cholesterol, has also been shown to alter the gut microbiota to provide a therapeutic advantage against radiation-induced injury in mice (Cui et al., 2019). Maintenance of a healthy gut microbiome appears to be essential in overcoming radiation-induced injury, as supported by studies that highlight the importance of this balance. It may be possible to repurpose existing products to modify the microbiome.

## Microbiome Biomarkers as Biodosimeters

In the case of a radiation mass casualty incident, H-ARS and GI-ARS subsyndromes will pose an immediate public health risk (Donnelly et al., 2010). The mean lethal radiation dose in humans that will kill 50% of those exposed within 60 days (LD_50/60_) is 3.25–4 Gy in the absence of supportive care but can be increased to 6–7 Gy with appropriate medical interventions (Waselenko et al., 2004). Consequently, effective triage of potentially exposed individuals in order to identify and separate those in need of immediate medical interventions (>2 Gy adsorbed dose) from the “worried well” (<2 Gy) requires a deployable biodosimetry method capable of making such distinctions so that limited medical resources can be used most efficiently (Dainiak, 2018).

In acute radiation exposure, it is possible that changes in microbial species, or metabolites released by them, can be used to assess dose received or the extent of radiation injury in a mass casualty scenario, particularly in easily accessible samples, such as feces or urine, but also in blood. As mentioned earlier, many bacterial species and microbiota changes in the skin (Plichta et al., 2017), vagina (Brotman et al., 2014; Bai et al., 2019), oral cavity (Vanhoecke et al., 2015; Zhu et al., 2017; Hou et al., 2018; Anjali et al., 2020; Nishii et al., 2020; Vesty et al., 2020), and GI of humans (Lam et al., 2012; Guo et al., 2020) are associated with disease severity and may even be predictive of pathogenesis. Along with the finding that some radiation-induced microbiota changes are persistent out to 6 months (Lam et al., 2012; Zhao et al., 2019; Anjali et al., 2020; Nishii et al., 2020), these data support the use of the microbiota as potentially stable biomarkers for radiation exposure and injury.

Biomarkers for triage, definitive dose, predictive biodosimetry, and/or to inform treatment decisions will be needed in a mass casualty radiation scenario. Researchers have found that microbial-derived metabolic products in fecal samples were modulated in a dose- and time-dependent manner following irradiation reflecting microbiota family-level changes in rodents (Lam et al., 2012; Goudarzi et al., 2016) and NHPs (Pannkuk et al., 2017; Pannkuk et al., 2019). The feasibility of using the GI microbiome and related metabolites as biodosimeters for early triage are currently being researched (Cai et al., 2020). More content on the state of the science for metabolomics in radiation injury have been reviewed elsewhere (Ó Broin et al., 2015; Satyamitra et al., 2020). While many promising approaches (cytogenetic and multiple “omics” approaches) are currently under investigation to identify dose-dependent biomarkers with the potential to provide rapid field-deployable biodosimetry tests, as of the writing of this review, no FDA-cleared devices are available. Although the field is in its infancy, these data suggest that the microbiome can be a powerful tool for radiation biodosimetry.

## Conclusion

Undoubtedly, the human microbiome is complex and varies based on its location, but regardless, it is necessary to maintain organ, tissue, and immune homeostasis. When the delicate balance of commensal bacteria is disrupted, it can result in a perturbation of the resident microbiota and wreak havoc on the host. Of particular interest for this review is the effect of ionizing radiation on the GI, lung, and skin microbiomes. Radiation not only changes the flora in these and other systems but also causes a breakdown of the epithelial barrier integrity, affecting the ability of the GI tract, lung, and skin to protect the host from invasive pathogens. Given the serious impact radiation has on these environments, it is imperative that treatment options or MCMs that can restore the human microbiota or provide an advantage under these harsh conditions continue to be explored.

Understanding the essentials of what is needed to support a healthy microbiome niche can help provide insight about key metabolites and molecular signatures that could be used as predictive biomarkers or developed into drugs to restore homeostasis. This knowledge can also be harnessed to take advantage of the microbes and develop microbial-mediated drugs to target a particular niche. Overall, the wealth of knowledge about the microbiome continues to grow, and its potential as a target for development of MCMs and/or identification of biomarkers of radiation damage continue to be discovered, with many areas yet to be explored.

## Footnotes

1
https://www.avidityscience.com/media/wysiwyg/4230-MI4179_-_Drinking_Water_Acification.pdf.

2
www.clinicaltrials.gov.
